# Self-perceived burden in elderly patients with chronic co-morbidities: a latent profile analysis

**DOI:** 10.3389/fpsyg.2025.1601198

**Published:** 2025-10-06

**Authors:** Qiqi Chen, Yan Jia, Hui Li, SiLu Lv, Tianjiao Du, Achong Feng

**Affiliations:** ^1^Department of General Surgery, The Fifth People's, Hospital of Ningxia Hui Autonomous Region, Shizuishan, Ningxia, China; ^2^Ningxia Fifth People's Hospital Dawukou Hospital, Shizuishan, Ningxia, China; ^3^Department of Nursing, The Fifth People's Hospital of Ningxia Hui Autonomous Region, Shizuishan, Ningxia, China; ^4^Department of Nursing, Jiangning Clinical Medical School of Kangda College of Nanjing Medical University, Nanjing, Jiangsu, China; ^5^Department of Nursing, The Affiliated Jiangning Hospital with Nanjing Medical University, Nanjing, Jiangsu, China; ^6^The First Clinical Medical College, Ningxia Medical University, Yinchuan, China; ^7^School of Nursing, Shanxi Medical University, Taiyuan, Shanxi, China

**Keywords:** older adult patients with chronic co-morbidities, self-perceived burden, latent profile analysis, medication literacy, technology anxiety, psychology

## Abstract

**Objective:**

This study aims to identify latent categories and characteristics of self-perceived burden among elderly patients with chronic comorbidities through latent profile analysis, and to analyze influencing factors across different latent categories.

**Methods:**

This study adopted a convenience sampling method, this research enrolled 632 hospitalized elderly patients with chronic comorbidities as study participants from January to April 2024. Data collection utilized surveys including general information questionnaires, the self-Perceived burden scale, the medication knowledge scale for elderly chronic disease Patients, and the technophobia scale. Latent profile analysis was conducted to characterize the self-perceived burden among respondents, with one-way ANOVA and logistic regression analysis employed to explore influencing factors across different categories.

**Results:**

This study collected 611 valid questionnaires. Based on potential profile analysis, the self-perceived burden of elderly patients with chronic diseases was categorized into three groups: moderate, low, and high symptom groups, accounting for 43.54, 36.49, and 19.97%, respectively. Univariate analysis revealed that factors such as status of occupational, personal monthly income, caregivers, residential mode, medical insurance type, daily exercise duration, course of disease, times of hospitalizations, self-rated sleep status, medication literacy and technology anxiety impact the self-perceived burden of chronic comorbidities across different categories.

**Conclusion:**

Older adult patients with chronic comorbidities demonstrated heterogeneity in latent profiles of self-perceived burden, with those experiencing moderate burden accounting for the highest proportion. Medication literacy and technology-related anxiety significantly influenced self-perceived burden. Healthcare professionals should develop targeted health education programs and early intervention strategies to reduce patients’ self-perceived burden levels.

## 1 Introduction

Population aging has led to increased incidence and prevalence of age-related diseases, resulting in a higher proportion of individuals with multiple chronic conditions (Gupta et al., 2022), and exacerbating the phenomenon of multimorbidity (Jin et al., 2025). In China, the population aged 60 and above has reached 264 million, accounting for 18.70% of the total population (Statistics, 2022). Among these, the prevalence of two or more chronic diseases among rural elderly in China stands at 44.46% (Wei et al., 2019). Patients with chronic disease comorbidities are more prone to developing negative psychological states during long-term treatment (Dong, 2022). Consequently, to improve treatment outcomes for these patients, their mental health status has garnered increasing attention from researchers.

Self-perceived burden refers to the psychological feeling experienced by patients who due to their illness and care needs, become increasingly dependent on family members for nursing, emotional, and financial support, resulting in a sense of being a burden to their families (Yeung et al., 2019). Under the influence of self-perceived burden. Research on influencing factors has revealed that social elements such as financial burden (Zhang et al., 2024a, 2024b), average household monthly income, marital status (Jiang et al., 2025), stigma score (Zhang et al., 2025c; Zhang et al., 2025b), family function (Cui et al., 2025), physical activity (Yang et al., 2025), along with psychological factors including the meaning of life and dignity in incapacitated patients (Rong et al., 2025), rumination, loneliness (Zhou et al., 2025), social support (Ren et al., 2024), and kinesiophobia (He et al., 2025) all impact an individual’s level of self-perceived burden. Several cross-sectional studies have indicated that the level of SPB in older adult hypertensive patients is associated with anxiety and medication adherence. Specifically, a lower perceived burden correlates with improved medication adherence, with gender, disease duration, and complications identified as independent influencing factors of SPB (Lin et al., 2018; Xiaodong, 2020). Furthermore, the self-perceived burden score of 59.46 ± 19.95 for patients with ovarian cancer falls within the moderate burden range, with self-perceived burden negatively correlating with family resilience and quality of life (Zhang et al., 2024a, 2024b).

Current studies on SPB predominantly focus on older adult patients with a single disease, primarily investigating the influencing factors pertaining to this group’s self-perceived burden. There is a notable lack of research addressing the self-perceived burden in older adult patients suffering from multiplechronic diseases. Moreover, existing studies on self-perceived burden predominantly focus on cancer patients, with limited research simultaneously examining multiple psychological factors in influencing factor analysis. Research findings indicate that older adult individuals with multiple comorbidities are more likely to concurrently experience depressive disorders and depressive symptoms (Zhong et al., 2020; Zhang et al., 2025a, 2025c). But there is also a lack of graded classification of self-perceived burden status based on severity scores. Intervention studies have demonstrated that acceptance and commitment therapy effectively alleviates fear of recurrence in young and middle-aged patients undergoing interventional therapy for liver cancer, thereby reducing perceived burden and enhancing nursing satisfaction (Song and Jun, 2021). Additional studies involving 60 hospitalized kidney transplant patients indicated that nursing interventions improved medication adherence and self-management capabilities, significantly reducing self-perceived burden and promoting patient prognosis (Hu et al., 2022).

In summary, accurately assessing self-perceived burden levels in older adult patients with chronic comorbidities, recognizing heterogeneity among different subgroups, and implementing effective interventions are particularly crucial for reducing individual burden. Latent profile analysis (LPA), as a statistical analysis method for classification, utilizes probability estimation and comparison within a probability model. This method centers on the individual and determines potential class variables by classifying individual potential features based on the results of external measurement items. It aims to elucidate the relationship between external continuous variables and categorize them into distinct classes according to their characteristics, thereby identifying independent categories (Bos et al., 2024; Vrieze, 2012). Currently, this statistical analysis method is increasingly applied across various research fields, including education, sociology, and psychology (Yang et al., 2024). This study employs latent profile analysis to explore latent categories and characteristics of self-perceived burden in older adult patients with chronic co-morbidities, while investigating influencing factors. This provides a theoretical foundation for developing targeted interventions and improving patient treatment outcomes.

## 2 Methods

### 2.1 Study design and participants

This study used a cross-sectional survey, This cross-sectional study followed the STROBE reporting guideline, and adopted convenient sampling method to select older adult patients with chronic comorbid diseases who were hospitalized in a tertiary hospital in Shizuishan City, Ningxia Hui Autonomous Region from January 2024 to April 2024 as the study objects.

The inclusion criteria were as follows:(1) patients≥60 years of age, (2) hospitalized patients, and (3) patients diagnosed by attending physicians with two or more diseases. Diseases considered for inclusion include, but are not limited to: cancer, hypertension, diabetes, coronary heart disease, cerebrovascular disease, hyperlipidemia, hyperuricemia, asthma, chronic obstructive pulmonary disease, gastroesophageal reflux, atrial fibrillation, thyroid disease and arthritis.

The exclusion criteria were as follows:patients who were participating in other clinical trials or nvestigations, and patients who temporarily dropped out due to sudden changes in their condition. According to the sample size calculation method for cross-sectional surveys (Ni et al., 2010), the sample size required for this study should be at least 5–10 times the number of independent variables.

The general data for this study included 17 independent variables, with the Chinese version of the Medication Literacy Assessment Scale contributing 4 independent variables, the Chinese version of the Self-Perceived Burden Scale contributing 1 independent variable, and the Chinese version of the Technical Anxiety Scale contributing 3 independent variables. This results in a total of 25 independent variables. Therefore, the formula for calculating the sample size is *N =* (17 + 4 + 1 + 3)*10 = 250. Considering an expected invalid questionnaire rate of 20%, the final sample size was calculated as *N =* 250/(1–0.20) ≈ 313. In this study, a total of 632 questionnaires were distributed, and 21 invalid questionnaires were excluded (17 with a single option selected and 4 with contradictory responses). Consequently, a total of 611 valid questionnaires were recovered, yielding an effective recovery rate of 96.68%.

### 2.2 Measures

#### 2.2.1 General information questionnaire

Through a comprehensive review of relevant literature and consultations with experts, this study developed a general information questionnaire comprising 17 items. These items encompass demographic and health-related variables, including gender, BMI, smoking history, drinking history, education level, marital status, occupational status, personal monthly income, family location, caregiver, living style, type of medical insurance, daily exercise duration, duration of illness, times of hospitalizations in the past year, self-rated sleep status, and age.

#### 2.2.2 Medication literacy scale for older adult patients with chronic diseases

The Medication Literacy Scale for Older adult Patients with Chronic Diseases was employed in this study (Zhao, 2023). This scale consists of 23 items categorized into four dimensions: information acquisition ability, drug knowledge reserve, communication and interaction ability, and critical ability. Each item is assessed using a 5-point Likert scale (1 = No, 5 = Totally OK). A higher score signifies improved medication literacy among older adult patients with chronic diseases. The reported Cronbach’s *α* coefficient is 0.958, indicating strong reliability. In the current study, the alpha coefficient of the scale was determined to be 0.956.

#### 2.2.3 Self-perceived burden scale

This study employs the Self-perceived Burden Scale (Simmons, 2007), which is specifically designed to assess the self-perceived burden experienced by patients with chronic diseases. The scale comprises a total of 10 items, each rated on a 5-point Likert scale (1 = never, 5 = always). The total score ranges from 0 to 50, with scores categorized as follows: 0–25 indicates a low level of burden, 26–33 indicates a medium level, and 34–50 indicates a high level of burden. A higher score reflects a greater perceived burden among patients. The scale demonstrates strong reliability, with a Cronbach’s alpha coefficient of 0.85; in this study, the Cronbach’s alpha coefficient was found to be 0.897.

#### 2.2.4 Technophobia scale

This study employs the Technophobia Scale to evaluate technology-related anxiety in the older adult population (Sang et al., 2022). The scale consists of 13 items, which are categorized into three dimensions: technology stress, technology fear, and security concerns. Each item is rated on a 5-point Likert scale (1 = completely inconsistent, 5 = completely consistent), with a higher total score indicating a greater level of technological anxiety. The overall Cronbach’s alpha coefficient for the scale is 0.867; however, in this study, the Cronbach’s alpha coefficient was found to be 0.932.

### 2.3 Data collection and quality control

After obtaining consent from the head nurses of each department, the researcher clearly articulated the purpose and significance of the study, along with key points for the respondents to consider. The questionnaire was distributed through an online platform.^1^ The head nurses shared the questionnaire link and informed consent with the nurses via their WeChat communication groups, enabling the study participants to complete the questionnaire independently online using electronic devices. In the design of the questionnaire, each question was marked as mandatory, and reminders were implemented for any unanswered items. Following the collection of the questionnaires, two members of the research team meticulously reviewed the gathered information and employed statistical software for data entry and analysis.

### 2.4 Statistical analysis

This study utilized MPLUS 8.3 software for latent profile analysis (LPA), while SPSS 27.0 statistical software was employed to conduct demographic variable analysis, analysis of variance, and logistic regression analysis. First, correlation analysis was performed using SPSS software to examine the means, standard deviations, and Pearson correlation coefficients of all variables. Subsequently, latent profile analysis (LPA) was employed to investigate the influencing factors of technology-related anxiety among older adult patients with chronic diseases.

The study selected eight explicit indicators: technology anxiety, Medication literacy, Status of occupational, Caregivers, Residential mode, Medical insurance type, Daily exercise duration, course of disease, Times of hospitalizations, Self-rated sleep status. By progressively increasing the number of latent categories, LPA models were constructed, and participant classification was determined based on analytical results. Finally, leveraging LPA outcomes, systematic analysis was conducted to examine differences in self-perceived burden across distinct patient sub types. In this study, the primary evaluation indicators included the Akaike Information Criterion (AIC), Bayesian Information Criterion (BIC), adjusted BIC (aBIC), and Entropy, which serve to assess the accuracy of classification. A smaller value of AIC, BIC, and aBIC indicates a better model fit, while Entropy ranges from 0 to 1, with values closer to 1 reflecting higher accuracy in model classification. The Lo–Mendell–Rubin adjusted Likelihood Ratio Test (LMRT) and the Bootstrapped Likelihood Ratio Test (BLRT) were employed to compare the fit of two adjacent models, with a *p*-value of less than 0.05 indicating that the k model was superior to the (k-1) model (Guo et al., 2024). After determining the optimal curve model, SPSS27.0 software was utilized for statistical analysis. The data in this study followed a normal distribution. Measurement data are presented as mean±standard deviation, while count data are expressed as frequencies. Chi-square tests and multifactorial logistic regression analysis were employed to identify risk factors for latent self-perceived burden in older adult patients with chronic comorbidities, with *p* < 0.05 considered statistically significant.

## 3 Results

### 3.1 General information of the study subjects

This study collected 611 valid questionnaires. The demographic survey revealed 339 male participants and 272 female participants, The total numbers of participants for no education, primary school, junior high school, vocational or junior college, bachelor’s degree or above, and illiterate are 379, 172, 186, 145, 51, and 57 respectively, In the monthly income survey, There are 222 participants below 3,000 CNY, 305participants between 3,000 and 4,999 CNY, 79 participants between 5,000–10,000 CNY, and 5 people above 10,000 CNY. In the residential location survey, 497 participants resided in urban areas, 114 in rural areas. Regarding caregivers during hospitalization: care was provided by spouses for 332 participants, by adult children for 217 participants; In the living arrangements survey: 396 lived with spouses, 135 lived with adult children, 62 lived alone; In the daily exercise duration survey: less than 30 min: 318 participants, over 2 h: 26 participants. Regarding disease course: 191 patients had a course of less than 6 months, 86 patients had a course of 3–5 years, and 100 patients had a course exceeding 5 years. Annual hospitalization frequencies: 307 patients were hospitalized 1–2 times, while 60 patients were hospitalized more than 5 times, as shown in Table 1.

**Table 1:** Comparison of baseline data among the three profiles of elderly patients with Chronic co-morbidities (*n =* 611).

Variable	C1 (*n =* 266)	C2 (*n =* 223)	C3 (*n =* 122)	*χ*^2^/F	*p*
Age	64.34 ± 15.67	63.18 ± 14.27	62.81 ± 15.37	*F* = 0.573	0.564
Gender				*χ*^2^ = 0.097	0.953
Male	146 (54.88)	124 (55.61)	69 (56.56)		
Female	120 (45.11)	99 (44.39)	53 (43.44)		
BMI				*χ*^2^ = 4.511	0.341
<18.5	24 (0.90)	28 (12.56)	10 (8.19)		
18.5–24	130 (48.87)	91 (40.81)	55 (45.08)		
≥24	112 (42.11)	104 (46.63)	57 (46.72)		
Smoking history				*χ*^2^ = 0.292	0.864
Yes	93 (34.96)	83 (37.22)	43 (35.25)		
No	173 (65.04)	140 (62.78)	79 (64.75)		
Drinking history				*χ*^2^ = 2.135	0.344
Yes	95 (35.71)	84 (37.66)	53 (43.44)		
No	171 (64.29)	139 (62.33)	69 (56.56)		
Degree of education					
Primary school	75 (28.19)	58 (26.01)	39 (31.97)	*χ*^2^ = 12.582	0.127
Junior high school	86 (32.33)	65 (29.15)	35 (28.69)		
Vocational or junior College	49 (18.42)	68 (30.49)	28 (22.96)		
Bachelor’s degree or above	25 (9.39)	16 (7.17)	10 (8.19)		
Illiterate	31 (11.65)	16 (7.17)	10 (8.19)		
Marital status				*χ*^2^ = 10.553	0.103
Married	216 (81.20)	198 (88.79)	96 (78.69)		
Unmarried	18 (6.76)	7 (3.14)	6 (4.92)		
Widowed or widowed	26 (9.77)	12 (5.38)	16 (13.11)		
Divorce	6 (2.25)	6 (2.69)	4 (3.27)		
Status of Occupational				*χ*^2^ = 10.361	0.035
Employees	57 (21.42)	58 (26.01)	42 (34.43)		
Retired	183 (68.79)	137 (61.43)	64 (52.46)		
Unemployment	26 (9.77)	28 (12.56)	16 (13.11)		
Personal monthly income				*χ*^2^ = 10.297	0.113
Below 3,000 CNY	108 (40.60)	69 (30.94)	45 (36.89)		
3,000–4,999 CNY	131 (49.24)	112 (50.22)	62 (50.82)		
5,000–10,000 CNY	25 (9.39)	40 (17.93)	14 (11.47)		
Above 10,000 CNY	2 (0.75)	2 (0.89)	1 (0.82)		
Family location				*χ*^2^ = 3.952	0.139
City	213 (80.07)	190 (85.20)	94 (77.05)		
Rural areas	53 (19.93)	33 (14.80)	28 (22.95)		
Caregivers				*χ*^2^ = 12.675	0.048
Spouse	134 (50.37)	137 (61.43)	61 (50)		
Children	100 (37.59)	71 (31.83)	46 (37.70)		
Others	20 (7.51)	12 (5.38)	13 (10.66)		
Relatives	12 (4.51)	3 (1.34)	2 (1.64)		
Residential mode				*χ*^2^ = 13.441	0.037
Living with husband and wife	160 (60.15)	162 (72.65)	74 (60.66)		
Living alone	26 (9.77)	21 (9.42)	15 (12.29)		
Children living together	69 (25.93)	38 (17.04)	28 (22.95)		
Other	11 (4.14)	2 (0.89)	5 (4.10)		
Medical insurance type					
Employee medical insurance	140 (52.63)	141 (63.23)	53 (43.44)	*χ*^2^ = 14.295	0.006
Resident medical insurance	117 (43.98)	77 (34.53)	62 (50.82)		
Self-financing	9 (3.38)	5 (2.24)	7 (5.74)		
Daily exercise duration				*χ*^2^ = 12.708	0.048
Less than 30 min	130 (48.87)	112 (50.22)	76 (62.29)		
30 min − 1 h	95 (35.71)	72 (32.29)	29 (23.77)		
1–2 h	32 (12.03)	24 (10.76)	15 (12.30)		
More than 2 h	9 (3.38)	15 (6.73)	2 (1.64)		
Course of disease				*χ*^2^ = 36.686	<0.001
Less than 6 months	62 (23.31)	74 (33.18)	55 (45.08)		
6 months −1 year	49 (18.42)	44 (19.73)	15 (12.29)		
1–3 years	71 (26.69)	30 (13.45)	25 (20.49)		
3–5 years	45 (16.92)	26 (11.66)	15 (12.29)		
5 years and above	39 (14.66)	39 (17.48)	12 (9.84)		
Times of hospitalizations				*χ*^2^ = 18.555	0.005
0 time	42 (15.79)	49 (21.97)	29 (23.77)		
1–2 times	126 (47.37)	124 (55.61)	57 (46.72)		
3–5 times	72 (27.07)	32 (14.35)	20 (16.40)		
5 times or more	26 (9.77)	18 (8.07)	16 (13.11)		
Self-rated sleep status				*χ*^2^ = 19.160	<0.001
Very good	51 (19.18)	59 (26.46)	45 (36.89)		
General	157 (59.02)	135 (60.54)	56 (45.90)		
Relatively poor	58 (21.80)	29 (13.00)	21 (17.21)		
Medication Literacy	71.4 ± 17.62	77.5 ± 25.56	89.6 ± 22.57	*F* = 29.089	<0.001
Technical anxiety	30.4 ± 10.03	21.6 ± 10.18	38.6 ± 13.41	*F* = 101.880	<0.001

*n*: the figures refer to absolute frequencies, C1-medium-Self-perceived burden group; C2-low-Self-perceived burden group; C3-high-Self-perceived burden group.

### 3.2 Current status of the variables

In this study, the self-perceived burden score was 28.11 ± 8.81, the medication management literacy score was 77.29 ± 22.78, and the technology-related anxiety score was 28.85 ± 12.50. Significant positive correlations were observed between self-perceived burden and both medication management literacy (r = 0.184, *p* < 0.01) and technology-related anxiety (r = 0.550, *p* < 0.01) in older adult patients with chronic comorbidities. Moreover, a significant positive correlation exists between medication literacy and technology-related anxiety (r = 0.251, *p* < 0.01), as shown in Table 2.

**Table 2:** The means, standard deviations, and correlation coefficients of the main variables (*n =* 611).

Items		Score range	M ± SD	1	2	3
1	Self-Perceived Burden	14–46	28.11 ± 8.81	1		
2	Medication Literacy	23–115	77.29 ± 22.78	0.184^**^	1	
3	Technology anxiety	13–52	28.85 ± 12.50	0.550^**^	0.251^**^	1

M, Mean; SD, Standard Deviation. *P < 0.05, **P < 0.01.

### 3.3 Latent profile analysis

This study used 10 indicators of perceived burden as explicit variables. we gradually increased the number of latent classes from 1 to 5 in this study. When retaining three classes, the Akaike Information Criterion (AIC), Bayesian Information Criterion (BIC), and adjusted Bayesian Information Criterion (aBIC) all reached their lowest values, while the entropy value was the highest. This indicates superior classification accuracy of the model. Furthermore, both the Lo–Mendell–Rubin Likelihood Ratio Test (LMRT) and the Bootstrap Likelihood Ratio Test (BLRT) yielded significant results (*p* < 0.001). Consequently, we retained the 3-class solution as shown in Table 3. Data in Table 3 demonstrate that during the testing of 2 to 5 classes, the 3-class model outperformed other classification models based on information criterion evaluation. After comprehensive consideration of all factors, the 3-class model was ultimately selected as the optimal solution. The correct classification probabilities for each category were 97.6,98.3 and 98.3% respectively, validating the reliability of the classification and latent profile analysis, as shown in Table 4.

**Table 3:** Fit indices for five models using latent profile analysis (*n =* 611).

Profile	AIC	BIC	aBIC	Entropy	*p*		Class probability
					LMRT	BLRT	
1	19822.179	19910.481	19846.985	–	–	–	1.000
2	16028.019	16164.887	16066.468	0.948	*p*<0.001	*p*<0.001	0.992/0.008
**3**	**13952.483**	**14137.917**	**14004.576**	**0.957**	***p*<0.001**	***p*<0.001**	**0.4354/0.3649/0.1997**
4	13094.702	13328.702	13160.438	0.955	*p*<0.001	*p*<0.001	0.284/0.175/0.373/0.166
5	12426.561	12709.127	12505.941	0.954	*p*<0.001	p<0.001	0.171/0.261/0.310/0.160/ 0.094

The selected models AIC, BIC and ABIC are the information evaluation indexes; *p* − LMR and *p* − BLRT are the model fit test indexes. C1: medium-Self-perceived burden group; C2: low-Self-perceived burden group; C3: high-Self-perceived burden group. The bold values indicators of each dimension of the model when the latent profile analysis in this study is categorized into 3 classes.

**Table 4:** Probability of correct classification for each category (*n =* 611).

Profile	C1	C2	C3
C1	0.976	0.015	0.009
C2	0.017	0.983	0
C3	0.017	0	0.983

C1-medium-Self-perceived burden group; C2-low-Self-perceived burden group; C3-high-Self-perceived burden group.

Patients with C1 whose self-perceived burden score is between C2 and C3 are named as medium-Self-perceived burden group. Accounting for 43.54% (266 cases). Patients with C2 had the lowest self-perceived burden score, which was named low-Self-perceived burden group. Accounting for36.49% (223 cases). Patients with C3 had the highest self-perceived burden score, named as high-Self-perceived burden group, accounting for19.97% (122 cases), as shown in Table 1.

### 3.4 Univariate analysis of potential categories of self-perceived burden characteristics

Three latent categories of self-perceived burden in older adult patients with chronic diseases demonstrated statistically significant differences (*P*<0.05), caregivers, residential mode, medical insurance type, daily exercise duration, course of disease, times of hospitalizations, self-rated sleep status, medication Literacy, occupational status and technical anxiety, as shown in Table 4.

### 3.5 Multivariate logistic regression analysis of potential categories of self-perceived burden characteristics

This study employed statistically significant variables as independent variables, with the self-perceived burden categories of older adult chronic disease patients serving as the dependent variable. Influencing factors showing significant statistical differences in univariate analysis were incorporated into multivariate linear regression. Multifactorial logistic regression analysis was then conducted using the moderate self-perceived burden group as the reference category. The findings of this study indicate that compared with the reference group (C1), higher medication management literacy (OR = 1.022, *p* < 0.001, OR = 1.040, *p* = 0.001) and technology-related anxiety (OR = 0.918, *p* < 0.001, OR = 0.920, *p* < 0.001) were associated with increased likelihood of patients experiencing high self-perceived burden. Compared with the unemployed reference group, both employed and retired patients demonstrated significantly lower odds of developing low self-perceived burden (OR = 0.383, *p* = 0.026) (OR = 0.438, *p* = 0.026). Compared to the no-specific-insurance reference group, urban employee medical insurance participants showed a near-significantly higher likelihood of developing high self-perceived burden (OR = 3.307, *p* = 0.096). Patients hospitalized 1–2 times had a significantly increased likelihood of developing low self-perceived burden compared to the no-hospitalization reference group (OR = 2.543, *p* = 0.021) as shown in Table 5.

**Table 5:** Results of multinomial logistic regression analysis (*n =* 611).

Characteristics		C2 VS C1						C3 VS C1					
		*β*	*SE*	Wald *χ*^2^	*P*	95% CI		*β*	*SE*	Wald *χ*^2^	*P*	95% CI	
Intercept		−0.472	1.323	0.127	0.721			−4.624	1.336	11.986	0.001		
Medication Literacy		0.022	0.005	16.191	0.000	1.011	1.033	0.039	0.007	28.275	0.000	1.025	1.055
Technical anxiety		−0.085	0.011	64.680	0.000	0.899	0.937	0.038	0.012	10.187	0.001	1.015	1.064
Status of Occupational	Employees	−0.959	0.430	4.981	0.026	0.165	0.890	−0.122	0.481	0.064	0.800	0.345	2.274
	Retired	−0.824	0.371	4.947	0.026	0.212	0.907	−0.783	0.421	3.457	0.063	0.200	1.043
	Unemployment	Ref						Ref					
Caregivers	Spouse	0.935	0.796	1.381	0.240	0.536	12.114	0.903	0.911	0.981	0.322	0.413	14.715
	Children	1.048	0.802	1.708	0.191	0.592	13.734	1.689	0.908	3.461	0.063	0.914	32.113
	Others	0.359	0.858	0.175	0.675	0.267	7.690	0.940	0.942	0.996	0.318	0.404	16.216
	Relatives	Ref						Ref					
Residential mode	Living with husband and wife	1.433	0.970	2.183	0.140	0.626	28.077	0.300	0.856	0.123	0.726	0.252	7.223
	Living alone	1.490	0.973	2.343	0.126	0.658	29.887	0.395	0.844	0.219	0.640	0.284	7.762
	Children living together	1.090	0.998	1.192	0.275	0.420	21.049	0.219	0.903	0.059	0.808	0.212	7.302
	Other	Ref						Ref					
Medical insurance type	Employee medical insurance	−0.240	0.746	0.103	0.748	0.182	3.397	−1.196	0.719	2.764	0.096	0.074	1.239
	Resident medical insurance	−0.574	0.736	0.609	0.435	0.133	2.383	−0.773	0.709	1.189	0.276	0.115	1.852
	Self-financing	Ref						Ref					
Daily exercise duration	Less than 30 min	−0.438	0.555	0.621	0.431	0.217	1.917	0.351	0.947	0.137	0.711	0.222	9.085
	30 min − 1 h	−0.505	0.565	0.798	0.372	0.199	1.827	−0.034	0.959	0.001	0.972	0.148	6.333
	1–2 h	−0.546	0.614	0.789	0.374	0.174	1.932	0.504	0.988	0.260	0.610	0.239	11.480
	More than 2 h	Ref						Ref					
Course of disease	Less than 6 months	−0.441	0.355	1.544	0.214	0.321	1.290	0.405	0.478	0.715	0.398	0.587	3.829
	6 months −1 year	−0.575	0.355	2.624	0.105	0.280	1.128	−0.617	0.513	1.449	0.229	0.198	1.474
	1–3 years	−1.563	0.360	18.873	0.000	0.104	0.424	−0.004	0.465	0.000	0.993	0.400	2.477
	3–5 years	−0.912	0.380	5.756	0.016	0.191	0.846	−0.328	0.505	0.422	0.516	0.268	1.939
	5 years and above	Ref						Ref					
Times of hospitalizations	0 time	1.064	0.484	4.842	0.028	1.123	7.483	−1.301	0.527	6.095	0.014	0.097	0.765
	1–2 times	0.935	0.406	5.313	0.021	1.150	5.645	−1.113	0.431	6.665	0.010	0.141	0.765
	3–5 times	0.359	0.438	0.673	0.412	0.607	3.377	−0.969	0.461	4.413	0.036	0.154	0.937
	5 times or more	Ref						Ref					
Self-rated sleep status	Very good	0.171	0.361	0.224	0.636	0.585	2.405	0.241	0.406	0.353	0.552	0.575	2.819
	General	0.143	0.298	0.230	0.631	0.643	2.072	0.097	0.347	0.079	0.779	0.558	2.176
	Relatively poor	Ref						Ref					

C1: medium-Self-perceived burden group; C2: low-Self-perceived burden group; C3: high-Self-perceived burden group.

## 4 Discussion

### 4.1 Current status of the variables

This study surveyed 611 older adult individuals with chronic comorbidities, revealing an average self-perceived burden score of 28.11 ± 8.11 indicating a moderate to high level. This is slightly lower than findings in females with inflammatory bowel disease (Liu et al., 2024), potentially attributable to differences in study populations and disease courses. This study found that the high self-perceived burden group had a higher proportion of males than females, which may be attributed to men undertaking more predominant societal roles and pressures compared to women.

This study yielded the following findings regarding influencing factors of chronic disease comorbidities. First, among economic factors, status of occupational and medical insurance type were significant influencing factors for self-perceived burden in this population. This aligns with findings from Ye et al. (2025), as older adult individuals have limited income and unstable financial resources. Facing expenditures for healthcare and daily living often creates financial strain, leading them to perceive themselves as economic burdens to their families. On the other hand, when older adult individuals rely on their adult children for support, they may develop psychological guilt, thereby intensifying their self-perceived burden. Secondly, among personal factors, six variables: caregivers, residential mode, daily exercise duration, course of disease, times of hospitalizations, and self-rated sleep status significantly influence the self-perceived burden in older adult patients with chronic diseases. Specifically, older adult individuals cared for by spouses or adult children may experience heightened self-perceived burden due to concerns about becoming a burden or perceiving caregiving pressure. Particularly when caregivers have limited energy, older adult individuals are more prone to developing guilt (Chen et al., 2025). Older adult individuals living alone or with distant family relationships often experience loneliness and helplessness due to lack of emotional support, which amplifies negative perceptions of their dependency and increases psychological burden. Older adult people with insufficient exercise exhibit more pronounced physical decline. Activity limitations reinforce their “uselessness “(He et al., 2025), while the positive status of regular exercisers helps alleviate burden perception. Frequent hospitalizations due to recurrent illnesses and treatment pressures (Liu et al., 2025) lead older adult patients to develop pessimistic views about their health. Combined with concerns over medical expenses, this significantly elevates self-perceived burden levels—a finding consistent with multiple studies. Finally, this study innovatively identified that among psychological factors, Medication Literacy and Technical Anxiety also influence Self-perceived Burden in older adult patients with chronic diseases. This may occur because insufficient medication management literacy heightens fears of incorrect dosing or missed medications, or inability to manage complex treatment regimens, thereby fostering feelings of helplessness. Conversely, robust medication literacy enhances self-management confidence and alleviates psychological pressure. During interactions with intelligent medical devices or electronic health systems, older adult patients are more prone to develop anxiety. This technological apprehension may amplify fears that technical barriers could compromise treatment efficacy, thereby intensifying self-devaluation and exacerbating psychological burdens associated with dependency on others.

### 4.2 Correlations among self-perceived burden, medication literacy and technical anxiety

This study revealed significant positive correlations between self-perceived burden and both medication management literacy as well as technology-related anxiety among older adult patients with chronic comorbidities. Furthermore, a significant positive correlation was observed between medication literacy and technology-related anxiety. Analysis suggests two primary reasons: First, patients with higher medication literacy tend to focus more extensively on disease management details. Their heightened awareness of medication regimen complexities and potential side effects may foster excessive concern about personal health status and perceived potential burden on family members, consequently intensifying self-perceived burden. On the other hand, patients with high technology-related anxiety fear being unable to master intelligent medical devices or electronic health systems, worrying this may compromise treatment adherence or increase family caregiving burden, thereby reinforcing their perception of being a burden. Furthermore, the positive association between medication management literacy and technology-related anxiety suggests that patients with better disease management knowledge may be more inclined to adopt new technologies, yet their unfamiliarity with these technologies conversely heightens anxiety. This aligns with the findings of Ye et al. (2025) and Khattak et al. (2025).

### 4.3 Latent profile analysis of self-perceived burden among older adult patients with chronic co-orbidities

This study classified self-perceived burden among older adult patients with chronic diseases into three categories using Latent Profile Analysis (LPA), with specific scores as follows: the medium self-perceived burden group (C1) scored 29.76 ± 3.23, the low self-perceived burden group (C2) scored 19.04 ± 3.71, and the high self-perceived burden group (C3) scored 41.08 ± 4.08. Notably, the medium self-perceived burden group (C1) comprised the largest proportion with 266 individuals, indicating that a majority of older adult chronic disease patients experience moderate levels of self-perceived burden. This finding warrants heightened attention from both family members and healthcare professionals.

Furthermore, the multivariate linear regression results revealed that higher levels of medication management literacy and technology-related anxiety were associated with increased likelihood of patients experiencing high self-perceived burden. This phenomenon may occur because patients with advanced medication literacy tend to focus more intently on disease details, potentially developing psychological stress from concerns about medication complexity and treatment efficacy. Concurrently, patients with heightened technology-related anxiety fear being unable to operate intelligent medical devices properly, worrying this may compromise treatment outcomes. Both factors reinforce the perception of “being a burden,” thereby intensifying self-perceived burden. These findings align with Zhang’s research (Zhang et al., 2025a), which similarly indicates that anxiety exacerbates patients’ self-perceived burden.

In conclusion, considering the current status and influencing factors of self-perceived burden among older adult patients with chronic comorbidities, multi-dimensional interventions are required to effectively reduce this burden in older adult chronic disease patients. First, patients themselves should enhance medication literacy and echnological adaptability. Accessible formats like illustrated manuals and video tutorials should disseminate chronic disease medication knowledge to reduce helplessness stemming from lack of understanding’. Patients should be encouraged to actively express disease-related concerns to strengthen self-efficacy. Second, family members must proactively listen to patients’ psychological needs, avoiding excessive protection or blame that exacerbates guilt; this alleviates patients’ psychological burden of “being a burden on family.” Third, healthcare professionals should incorporate integrated “psychological-disease” assessments during clinical consultations, proactively inquire about patients’ anxieties regarding medication regimens and technology, provide tailored simplified medication plans, regularly organize peer support meetings to share successful management experiences, and alleviate loneliness and helplessness through peer support. Fourth, medical device manufacturers need to enhance age-friendly design in products to lower the technological barrier for older adult patients; concurrently develop simplified user guides for collaborative “family member-patient” use, thereby reducing sources of technology-related anxiety at the root. Through synergistic collaboration across four dimensions, comprehensive interventions are implemented from the perspectives of knowledge empowerment, emotional support, and technical adaptation to systematically reduce self-perceived burden among older adult patients.

## 5 Limitations

This study has the following limitations. Firstly, the convenience sampling method employed was limited to the Shizuishan urban area, resulting in a relatively small sample size that may introduce reporting bias. Additionally, the included influencing factors were insufficiently comprehensive. Future research could expand the sample selection scope and conduct multi-center studies. Secondly, the research design focused exclusively on chronic disease patients without analyzing the impact of specific disease types on self-perceived burden. Subsequent studies should incorporate multiple variables such as disease type, severity, and medical costs and different ages, to inform intervention research and provide guidance. Thirdly, The cross-sectional design prevents causal inferences about the relationships between influencing factors and SPB. Future research could benefit from incorporating machine learning and more diverse modeling techniques to enhance the model’s precision and explore dynamic changes in SPB over time. Finally, this study focused exclusively on older adult individuals aged 60 years and above with chronic comorbidities. Future research should conduct stratified and diversified studies to identify critical turning points for self-perceived burden across different age groups. Implementing phased targeted interventions and preventive measures can effectively alleviate psychological burden in older adult patients with chronic diseases.

## 6 Conclusion

This study employed latent profile analysis to systematically explore self-perceived burden levels among older adult chronic disease patients, utilizing medication literacy, technology-related anxiety levels, and sociodemographic data. The research revealed that self-perceived burden can be categorized into 3 types: low self-perceived burden, high self-perceived burden, and moderate self-perceived burden. The study further identified specific influencing factors across three dimensions: individual factors, economic factors, and psychological factors. Patients themselves, family members, healthcare professionals, and medical device manufacturers should thoroughly understand the individual characteristics of the older adult population, with particular focus on those experiencing moderate and high self-perceived burden, implementing targeted interventions to effectively reduce their burden levels.

## Data Availability

The raw data supporting the conclusions of this article will be made available by the authors, without undue reservation.

## Footnotes

^1^ https://www.wjx.cn

## Glossary

SPB (Self-perceived Burden): Self-perceived burden refers to the psychological feeling experienced by patients who due to their illness and care needs, become increasingly dependent on family members for nursing, emotional, and financial support, resulting in a sense of being a burden to their families.
LPA (Latent Profile Analysis): A statistical method for identifying unobserved (latent) subgroups in a population based on individuals’ responses to continuous observed variables, assuming distinct response patterns per subgroup.
AIC (Akaike Information Criterion): A model fit index that balances goodness-of-fit and complexity, calculated as −2log-likelihood + 2 k. Lower values suggest a better trade-off between fit and model parsimony.
BIC (Bayesian Information Criterion): A model selection criterion that penalizes complexity more than AIC, calculated as −2log-likelihood + k × ln(n). Lower BIC values indicate a more parsimonious and likely better-fitting model.
aBIC (Adjusted Bayesian Information Criterion): A modification of BIC that adjusts for sample size, providing a more reliable criterion for model selection, especially in moderate to large samples. Lower values are preferred.
LMRT (Lo–Mendell–Rubin Adjusted Likelihood Ratio Test): A hypothesis test comparing a k-class model to a (k − 1)-class model. A significant *p*-value (<0.05) indicates that the k-class solution provides a significantly better fit.
BLRT (Bootstrapped Likelihood Ratio Test): A resampling-based test that compares the fit of a k-class model with a (k − 1)-class model using bootstrap samples. A significant *p*-value supports the retention of the k-class solution.
